# Refining Care to Plan: Delivering Personalized Recommendations to Support Dementia Caregivers

**DOI:** 10.1093/geroni/igab046.2007

**Published:** 2021-12-17

**Authors:** Jinhee Cha, Colleen Peterson, Ashley Millenbah, Katie Louwagie, Zachary Baker, Christine Jensen, Joseph Gaugler

**Affiliations:** 1 University of Minnesota, University of Minnesota, Minnesota, United States; 2 University of Minnesota, University of Minnesota/Minneapolis, Minnesota, United States; 3 Riverside Center for Excellence in Aging & Lifelong Health, Williamsburg, Virginia, United States; 4 University of Minnesota, Minneapolis, Minnesota, United States

## Abstract

Caregivers of persons living with Alzheimer’s disease and Alzheimer’s disease-related dementias (PLWD; AD/ADRD) benefit from unique interventions to address their different needs. While information on which interventions best meet specific needs exists, less is known about how to match caregivers with those interventions. To address this research gap, we tested Care to Plan (CtP) within a large healthcare system. After care navigators guided caregivers through twenty CtP tailoring questions to identify caregivers’ greatest needs, the online tool provided the intervention type best suited to meet their needs, along with region-specific information on available programs. This mixed methods analysis evaluated the utility of the CtP tool with 20 family caregivers of PLWD after a 1 month follow-up. Most caregivers agreed that the CtP tool was helpful (85%) and would recommend the tool to other caregivers (90%). Caregivers also said they valued being able to discuss the CtP recommendations with the care navigator (95%). However, only 65% said they found services that met their needs or planned on using services recommended by CtP. Interview data indicate time constraints and restricted availability of resources due to COVID-19 precautions reduced caregivers’ abilities to pursue some recommendations. In addition, the stage of dementia experienced by their care recipient may explain why others found CtP less useful. However, these caregivers noted the potential utility of the resources for their future care planning needs. A larger evaluation of the CtP tool within the healthcare system is ongoing.

